# Aequalis Humeral Head Resurfacing in Glenohumeral Arthritis at a Minimum Followup of 2 Years

**DOI:** 10.1155/2013/541389

**Published:** 2013-06-11

**Authors:** Roshan Raghavan, Amitabh J. Dwyer, Andrew F. W. Chambler

**Affiliations:** Yeovil Elbow and Shoulder Service, Department of Orthopaedic Surgery, Yeovil District Hospital, Higher Kingston, Yeovil, Somerset, BA21 4AT, UK

## Abstract

*Aim*. To evaluate results of Aequalis humeral head resurfacing in patients with end-stage glenohumeral arthritis at a minimum followup of two years. *Patients and Methods*. Twenty-one consecutive patients underwent humeral head resurfacing hemiarthroplasty between 2007 and 2009. Three patients did not fulfill the inclusion criteria. 18 patients with mean age of 75.1 years (range 58–91 years) and a mean duration of preoperative symptoms of 33.6 months (range 6–120 months) were analyzed. Patients' self-reported Oxford shoulder score (OSS) was collected prospectively and was used as an assessment tool to measure final outcome. *Results*. The mean initial OSS was 15 (range 3–29). The score improved by an average of 19.5 points at a mean followup of 36.3 months (range 24–54 months) to reach a mean final OSS of 34.5 (range 6–47). The improvement of OSS was highly significant with a two-tailed *P* value less than 0.0001. The overall patient satisfaction was 94%. *Conclusion*. This study demonstrates Aequalis shoulder resurfacing hemiarthroplasty as a reliable procedure, away from its originating center, for improvement of shoulder function as shown by the patients' self-reported outcome score (OSS) in end-stage glenohumeral arthritis at a minimum followup of 2 years.

## 1. Introduction

Humeral head resurfacing was proposed as a treatment for glenohumeral arthrosis in an attempt to preserve the original anatomy and avoid humeral head resection. Preservation of humeral head maintained the native inclination, offset, head shaft angle, and version of humerus [1–3]. Other advantages include a shorter operating time, reduced blood loss, and fewer complications [4]. Another advantage is that, unlike stemmed implant, there is no need for a straight humeral canal to accommodate a long stem [4]. Resection of bone is minimal and bone cement is not used. This allows easier later revision to a conventional total shoulder arthroplasty, if required [1, 2]. It is an attractive option in both the old and the young patients [4, 5]. The disadvantage of resurfacing is the limited exposure to glenoid when wanting to perform a total shoulder resurfacing arthroplasty, but this does not affect when resurfacing the humeral head alone. 

The primary aim of our study was to report the results of humeral resurfacing arthroplasty in a consecutive series of patients at a district general hospital practice.

## 2. Patients and Methods

Twenty-one consecutive patients underwent shoulder resurfacing (Aequalis, Tornier, USA) between October 2007 and November 2009 for symptomatic end-stage glenohumeral arthrosis. Clinical examination and radiographic evidence were used as the benchmark for diagnosis. Patient demographics, duration of surgery, intraoperative findings, and complications were prospectively recorded. Only patients with a minimum followup of two years were included for analyses. Of the 21 patients, one patient had worsening of symptoms and underwent revision to a stemmed total shoulder replacement in less than 2 years, one patient died of unrelated cause, and in one patient the humeral head collapsed at the time of impaction of the definitive component and therefore was converted to a stemmed hemiarthroplasty intraoperatively. Therefore 18 patients with a mean followup of 36.3 months (range 24–54 months) were included in the study. There were 6 men and 12 women with a mean age of 75.1 years (range 58–91 years, 95% CI of the SD was 70.8 to 79.4), and there were 7 right and 11 left shoulders (Table 1). The mean duration of preoperative symptoms was 33.6 months (range 6–120 months, 95% CI of the SD was 22.6 to 45.2).

All patients were operated on in the beach chair position under general anaesthesia and an interscalene nerve block. The shoulder was exposed by a deltopectoral approach with the upper third of subscapularis and the joint capsule reflected in one layer. The inferior capsule was released of the humeral neck whilst protecting the axillary nerve. The humeral head was exposed by external rotation and adduction of the arm. The cartilage of humeral head and glenoid were inspected for wear, and osteophytes were excised from the head of the humerus. The rotator cuff was inspected for its integrity, and either a normal appearance or partial tear was recorded. Any inflammatory pathology of the rotator interval, integrity of the labrum and the long tendon of the biceps brachii and loose bodies in the inferior recess were recorded. The size of the humeral head was measured with the sizing guide. A guide wire was then introduced in the centre of the humeral head which was reamed to the measured size, and trial component inserted after a cruciate keel made a foot anchor in the cancellous bone of the humeral head. Soft tissue releases were undertaken, and stability and range of movement of the shoulder were assessed with trial implant in situ. Definitive component was then impacted. Subscapularis tendon and joint capsule were repaired using the tendon-to-tendon technique. The operation was performed by the senior author or by an orthopaedic trainee under direct supervision of the senior author. The mean duration of anaesthesia that included surgical time was 112 minutes (range 75–150 minutes, median 120 minutes). The postoperative regime was the same for all patients. This included monitoring of postoperative pain and neurovascular status, two further doses of IV cefuroxime 1.5 gm each at 8 and 16 hours, a check X-ray of the shoulder's anteroposterior and lateral views, a sling to be worn for comfort, no external rotation beyond neutral for 3 weeks and no active external rotation for 6 weeks, physiotherapy advice at discharge, and a physiotherapy followup at 3 weeks postoperatively. Anteroposterior, axillary, and lateral radiographs were taken before and after the operation.

Clinical outcome of the operation was assessed by patients' self-reported Oxford shoulder score (OSS) for pain. This consisted of 12 questions involving activities of daily routine. It has a best possible score of 48 and the worst score of 0 [6, 7]. The OSS data was collected prospectively before the operation and compared at final followup. Paired two-tailed *P* value was calculated to assess improvement in the outcome of the procedure, and a value of <0.05 was considered statistically significant. OSS is a patient-reported outcome measure, and its reliability has been validated against constant shoulder score, SF36 [6, 8, 9], western Ontario rotator cuff index (WORC), and Shoulder Pain and Disability Index (SPADI) [10]. Patient satisfaction questionnaire was also completed at final followup.

## 3. Results

The median duration of hospital stay was one day (mean 2 days, range 1–8 days). One patient complained of chest pain postoperatively and stayed for 8 days in the hospital. She was diagnosed to have a triple vessel coronary disease, which delayed her discharge due to investigative procedures. She later underwent a coronary artery angioplasty. The pre-operative mean OSS of 15 (range 3–29, 95% CI of the SD was 10.6 to 19.4) improved by 19.5 points to a final mean OSS of 34.5 (range 6–47, 95% CI of the SD was 29.3 to 39.7). The improvement of OSS was highly significant with a two-tailed *P* value less than 0.0001 and 95% confidence interval of this difference from 14.2 to 24.7.

15 patients (83.3%) had greater than 11-point improvement of OSS (Table 2). One patient developed adhesive capsulitis and his pre-operative OSS of 18 declined to 15 at 54 months followup. This patient then underwent arthroscopic capsular release; however, he did not respond to further OSS questionnaires. Two patients each had 3 and 4 points improvement of OSS (Table 2). One of them had radiating pain from cervical spondylosis and was referred to pain clinic for management of her symptoms. No identifiable cause for the lack of greater than 4 points improvement of OSS was found in the other patient. The overall patient satisfaction rate was 94.5%. Apart from one patient, the remaining 17 patients were very or fairly pleased with the operation and, if they could go back in time, they still would choose to have the same operation. 

Of the excluded patients, one had an intraoperative collapse of the humeral head while impaction of the definitive implant and therefore was converted to a stemmed hemiarthroplasty. One patient had poor initial result from the resurfacing procedure and was revised to total shoulder replacement within the first two years. He had an initial OSS of 10 that only improved to 11 after resurfacing. X-rays at 10 months were suggestive of glenoid erosion. He made a significant improvement to a final OSS of 30 at one year after total shoulder replacement. 

None of the patients were lost to followup. There were no wound healing problems, infection, deep vein thrombosis, pulmonary embolism, and neurovascular deficits.

## 4. Discussion

Over the past twenty years, shoulder resurfacing arthroplasty has gained popularity as an alternative to conventional shoulder arthroplasty for the treatment of glenohumeral arthropathy [11]. The potential advantages of humeral resurfacing, as compared with conventional shoulder arthroplasty, that include minimal bone resection, a short operative time, low prevalence of humeral periprosthetic fractures, maintenance of head shaft angle, and an ease of revision to a conventional total shoulder replacement, if needed, are well documented [1, 2, 11]. Outcomes of various surface replacement arthroplasty designs have been comparable with those of arthroplasties with a stemmed prosthesis in numerous short and mid-term follow-up studies [1, 2, 11]. These include the Copeland surface replacement prosthesis (Biomet), the Durom cup (Zimmer), Total Articular Replacement Arthroplasty prosthesis (DePuy Orthopaedics [Warsaw, IN, USA], and Howmedica [Rutherford, NJ, USA]) [1, 2, 12, 13]; however, our study presents results of the Aequalis shoulder resurfacing implant (Tornier) which have not been published in the literature. Our consecutive series of 18 patients with end-stage glenohumeral arthritis showed a mean 19.5 points (range −3 to 36) improvement in OSS at a mean followup of 36.3 months. Most (15/18) patients had an improvement of 11 points or more. These results are comparable to change in the Oxford shoulder score of a mean 25 points (range 42 to 17) with TESS anatomic prosthesis [14]; however, this study used the older version of OSS [8, 9] with best possible score of 12 points and worst score of 60 points. 

Most patients (94.5%) were very or fairly pleased with the operation similar to the 90% satisfaction rate reported by Huguet et al. [14]. Our overall complication rate was 14.3%; however, this can partly be attributed to the initial learning curve rather that to the design or performance of the implant. The reasons for failure include poor selection of a patient where the humeral head collapsed intraoperatively at the time of impaction of the definitive prosthesis. Failures to appreciate glenoid wear preoperatively, which rapidly progressed after resurfacing. This patient had 19-points improvement after revision to a total shoulder replacement. And one patient who developed adhesive capsulitis, unrelated to the surgical procedure, presented with a fall in OSS score by three points and underwent arthroscopic capsular release. Our consecutive series of patients shows the good results through historical analysis of the prospectively collected data with no loss to followup. Our study of this implant is from a nonoriginating center of design and production, and to date the results of this implant have not been reported in the literature. The results show that it is comparable with previously published studies on other resurfacing implants [1, 2, 14].

The limitations of our study are due to smaller number of patients and early followup. Long-term results of this implant are not yet known. However, given the available data, our study shows very good results with Aequalis shoulder resurfacing prosthesis. It was not the purpose of this study to compare shoulder resurfacing with stemmed shoulder replacements, and no overall consensus was reached favoring one over the other. This may need a prospective, randomized, long-term study in the future.

## Figures and Tables

**Table 1:** Spreadsheet.

No.	Age	Sex	Side	Length of GA in minutes	Hospital stay in days	Followup in months	Preoperative OSS	Final OSS	Change in OSS	Complication
1	80	M	L	120	1	24	27	38	11	Nil
2	82	M	R	90	2	32	29	47	18	Nil
3	83	F	R	120	2	39	3	6	3	Nil
4	80	F	L	120	1	37	14	41	27	Nil
5	63	M	R	120	1	49	16	46	30	Nil
6	69	M	R	120	1	40	11	35	24	Nil
7	78	F	L	90	1	26	29	40	11	Nil
8	77	F	R	105	2	40	3	27	24	Nil
9	82	F	L	90	4	26	15	30	15	Nil
10	64	F	L	150	2	48	27	31	4	Nil
11	91	F	L	120	2	33	19	42	23	Nil
12	80	F	R	120	4	50	8	36	28	Nil
13	80	F	L	120	8	30	4	40	36	Nil
14	72	F	L	75	1	26	6	35	29	Nil
15	58	M	L	120	1	29	9	32	23	Nil
16	64	M	L	120	1	54	18	15	−3	Adhesive capsulitis
17	72	F	R	105	1	36	10	36	26	Nil
18	78	F	L	120	1	34	22	44	22	Nil

**Table 2:** Change in Oxford shoulder score.

Decrease in OSS	Number of patients *n* = 1
−3	1

Improvement in OSS	Number of patients *n* = 17

0–4	2
5–10	0
11–20	4
21–30	10
31–40	1
